# Salmagundi

**Published:** 1888-02

**Authors:** 

**Affiliations:** Cincinnati


### SALMAGUNDI.

EDITED BY E. G. BETTY, D.D.S.

MISSISSIPPI VALLEY DENTAL ASSOCIATION.—The coming meeting of this time-honored association promises to be one of the best that has ever been held, and we are glad of it, for the reason that it stirs up the folks about this neck of the woods for several months afterwards. The program, published upon another page of this issue, contains such a variety of material that none can fail to find something of interest and value to himself personally. In conning over the topics named we fail to discover any mention of, or paper upon the prize subject, viz: Salivary Calculus. This may or may not be due to the befogged condition of the committee, or rather, more technically speaking, the petrifaction of the products of putrefaction, the subject itself suggesting such an inference.

However that may be, we can promise to our readers an interesting report, and to those who treat themselves by attending, a glorious good time with many nuggets of gold to store away in their intellectual sox.

CINCINNATI, January 18, 1888.

*Editor Salmagundi:*—

DEAR SAL—Don't forget to boom the coming meeting of the Mississippi Valley Association of Dental Surgeons. The very name smacks of paleontolgy—and the society, though full of interesting specimens of fossils, offering rare opportunity for study in this line, presents the most remarkable feature of the age in this very respect; for they are *aninated* fossils—fossils which breathe, and talk, and write on modern topics with the fluency and spiciness of more recent biological specimens. Just imagine a trilobite waking up—its tissues gradually changing from *petrifaction* to the softer conditions liable to *putrefaction*, its heart beginning to throb, its blood to circulate, its wonderful eye to brighten up and wink at you—just imagine this creature then suddenly humping itself in search of a lunch, and beginning to look into the latest methods of the living creatures of to-day; nosing around among the bacteria and trying to classify them; smelling out the best antiseptics and trying to find out the best ways of preventing the young of this age from getting into the antiquated ways of his age. Why the thing becomes one of the most wonderful shows of the world right away! don't it? Well, that is what we can show you at the next meeting of the Mississippi Valley Association of Dental Surgeons. Give me a live fossil every time, instead of an uncertain microbe! A microbe with no definite pedigree; a microbe who has got to establish himself in life, and of whom we know nothing as yet. The fossil is always there, but where sometimes is the microbe?

Yours, MICRO-COCCI.

---

NEURALGIA—BELLADONNA AND NUX VOMICA.--The practitioner frequently meets with cases of neuralgia of the head or face, associated with obstinate constipation in the female. We may secure temporary ease from the neuralgia by the use of one or the other of the numerous so-called remedies for that condition, but the constipation persists; or we may overcome the constipation for a while by means of laxatives, aperients, etc., but the neuralgia still comes and goes. The patient is wearied by the continually recurring pain, and often depressed below par by the treatment of the constipation. In such cases I have found a quarter of a grain each of extracts of belladonna and nux vomica, given nightly, in a pill, to render great service. One lady states she has not been so long--one month—free from neuralgia in years. LOUIS LEWIS, M.D.

---

BORACIN--Is said to consist of boric acid, glycerine, methyl salicylate, menthol, thymol, and eucalyptol. It is used in solution as a wound dressing; as a thick paste, it is applicable to ulcers; as suppositories made with 45 per cent. of glycerin, it may be used in treating chronic leucorrhea, or to exterminate rectal parasites; and as an ointment it is a remedy for eczema.--*Am. Druggist.*

DISEASE GERMS.—"Every specific disease is due to the influence of a distinct morbid substance on some parts at which the characteristic signs of the disease can be and are manifested. Two conditions must coincide in each: the one general or diffused in a morbid material in the blood; the other local, in some part with which this material produces disease. By this morbid substance I mean those changes produced directly or indirectly by a distinct species of minute parasite, a microbe, a bacillus, or some other vegetable of lowest organization, yet specific. I believe that micro-parasites, or substances produced by them will eventually be found in essential relation with cancers."

SIR JAMES PAGET.

NEURALGIA REMEDY.—Dr. John T. Metcalf says (*Boston Medical and Surgical Journal*) that the following formula was learned by him from one of his patients whom he sent to Cuba with the hope that a change of climate would afford relief from sciatica. A French physician, who there attended him, used this remedy with the best results, and Dr. Metcalf has tried it so often with success, that he speaks of its value with great confidence: Equal parts of the tinctures of aconite root, colchicum seeds, belladonna, and cimicifuga. Six drops to be taken every six hours until relief is felt. The doctor says, that "as an internal remedy it is worth all others put together of which I have knowledge."—*Med. World.*

PAINLESS TOOTH EXTRACTION.—Dr. Bontemps addresses the following note to the *Journal of the American Society of the Limoges*: The extraction of teeth, more especially of large molars, may be effected, positively without pain, by binding the key-bit with a little wadding, maintained in position by a small linen band, and dropping on to it two or three drops, not more, of anesthetic chloroform. The pressure of the instrument is not felt, and the patient, while sensible that the tooth is being withdrawn, experiences no pain whatever. This method, so simple and effectual, is without danger or inconvenience, owing to the minute quantity of chloroform required and the insignificant duration of its action. How ancient!

INCISIONS FOR "MUMPS."—Parotitis should be treated locally by cataplasms, fomentations, mercurial applications, blisters, leeches, ice, etc.; and by sulphide of calcium, gray powder, etc., internally, until it is evident that resolution will not occur; then surgical interference is called for, before fluctuation is absolutely detected, in order to prevent pus from burrowing through the external auditory canal, or in the deeper regions of the neck. Incision is especially indicated when the swelling and edema are extreme, to avoid compression of the deep veins and consequent gangrene.

One bold incision should be made, parallel with the maxilla, towards the posterior part of the gland, so as to avoid the facial nerve. The fascia should be divided with a bistoury, and a small drainage-tube left in the wound. A mild antiseptic fluid may be injected through this tube, and the wound covered with an antiseptic dressing.—*Exchange.*

